# Editorial: Intracranial atherosclerotic disease: epidemiology, imaging, treatment and prognosis, volume II

**DOI:** 10.3389/fneur.2023.1310281

**Published:** 2023-11-21

**Authors:** Xinyi Leng, Shyam Prabhakaran, Jin Soo Lee, Alex Abou-Chebl, David S. Liebeskind

**Affiliations:** ^1^Department of Medicine and Therapeutics, The Chinese University of Hong Kong, Hong Kong, Hong Kong SAR, China; ^2^Department of Neurology, University of Chicago, Chicago, IL, United States; ^3^Department of Neurology, Ajou University School of Medicine, Ajou University Medical Center, Suwon-si, Republic of Korea; ^4^Department of Neurology, Henry Ford Health System, Detroit, MI, United States; ^5^Department of Neurology, Neurovascular Imaging Research Core and UCLA Stroke Center, University of California, Los Angeles, Los Angeles, CA, United States

**Keywords:** stroke, intracranial atherosclerotic disease, imaging, endovascular treatment, Stenting

Intracranial atherosclerotic disease (ICAD) is an important cause of ischemic stroke and transient ischemic attack (TIA) worldwide, particularly in Asian populations (1). The risks of stroke relapse and deaths have been declining in the last two decades, with more effective secondary stroke prevention strategies including dual/mono antiplatelet and high-intensity statin treatment, stringent vascular risk factor management, and lifestyle modifications (2). For instance, the risks within 1 year were respectively 23% and 18% in medically treated patients with symptomatic ICAD of 70–99% luminal stenosis, in the WASID (Warfarin Aspirin Symptomatic Intracranial Disease) (3) and SAMMPRIS (Stenting and Aggressive Medical Management for Preventing Recurrent Stroke in Intracranial Stenosis) (4) trials conducted over 20 and 10 years ago, which was down to around 8% in counterparts in the most recently published CASSISS (China Angioplasty and Stenting for Symptomatic Intracranial Severe Stenosis) trial (5). Yet, the residual risk is not negligible, which calls for more accurate risk stratification of the culprit lesion and the affected patient, as well as more effective treatment of the disease. Following a collection of ICAD studies published in 2020–2021 (Volume I) (6), this second volume of the Research Topic includes a review article on imaging methods/markers for ICAD and atherosclerosis in other vascular beds and four original research articles on imaging and treatment of ICAD and ICAD-related stroke/TIA.

In a review article, He et al. provided a comprehensive review of widely used and emerging imaging techniques and relevant imaging markers to assess the vessel lumen, vessel wall and plaque, and focal hemodynamics in the presence of atherosclerosis in various vascular beds, including mostly the coronary, carotid and intracranial arteries, after a summary of the pathophysiology of atherosclerosis, features of culprit plaques (in comparison to asymptomatic plaques) and distribution of intracranial and extracranial plaques. Among the emerging imaging techniques, 3D pointwise encoding time reduction magnetic resonance angiography (PETRA-MRA) may yield higher accuracy in determining the degree of luminal stenosis and lesion length in ICAD, as compared with 3D time-of-flight MRA and computed tomography angiography, using digital subtraction angiography as the reference standard, according to Niu et al., in a single-center, prospective study of 52 patients with 90 intracranial stenoses. Yet, on top of the degree of luminal stenosis that has been used as a single criterion to gauge the severity of ICAD in current practice, a combination of other imaging methods and consideration of plaque characteristics, hemodynamics and other imaging features (e.g., infarct topology) could aid more accurate risk stratification of ICAD and ICAD-related strokes, which is a vastly heterogeneous disease (7).

Drawing on the format of Volume I of this Research Topic (6), this second volume includes a study on acute endovascular treatment (EVT) of large vessel occlusion (LVO) due to ICAD. Based on secondary analysis of the DIRECT-MT (Direct Intra-arterial Thrombectomy in order to Revascularize Acute Ischemic Stroke Patients with Large Vessel Occlusion Efficiently in Chinese Tertiary Hospitals: a Multicenter Randomized Clinical) trial, Li et al. developed a predictive model based mostly on basic clinical and simple imaging variables, for early diagnosis of ICAD-related LVO vs. other etiologies. However, the authors acknowledge the unsatisfactory sensitivity (69%) and specificity (74%) of this model for predicting ICAD-related LVO in external validation, partly due to the small proportion (7%) of patients with ICAD-related LVO and the around 40% of undetermined etiology of LVO in the development cohort (i.e., the DIRECT-MT cohort) (8). Further work is needed to develop more accurate predictive models for such purposes, which may aid in clinical decision-making in EVT treatment for acute LVO.

Despite neutral findings of stenting vs. medical therapy for preventing stroke and death in high-grade, symptomatic ICAD patients in the SAMMPRIS (4) and CASSISS (5) trials, it may be worth testing the safety and efficacy of angioplasty with or without stenting in selective patients based on hemodynamics and/or the thromboembolic potential (7). Several articles in Volume I of this Research Topic (6) have discussed the risks of complications, stroke, TIA, and death after angioplasty ± stenting therapy in symptomatic ICAD patients. In Volume II, there are two articles discussing factors that may affect outcomes of stenting therapy in these patients. Zhang et al., reported a non-linear relationship between lesion length and risk of recurrent ischemic stroke or TIA pertinent to the same arterial territory, from 1 month after stenting to the end of the follow-up period (median follow-up 25 months). A longer lesion was associated with an increased risk of recurrent ischemic stroke/TIA in those treated with balloon-expandable or self-expanding stent when the length was <9.00 mm, while no significant relationship was found when the length was >9.00 mm in those treated with self-expanding stent. In another study by Liu et al., the stent shape (inner- or outer-narrowed, or inner- and outer-enlarged shapes) seems to be able to affect distributions of focal wall shear stress and low-density lipoprotein filtration rate, based on computational fluid dynamics simulations of idealized artery models and patient-specific models with high-grade ICAD. These may affect plaque growth and risks of in-stent restenosis and recurrent ischemic events, which, however, cannot be answered in this cross-sectional study. In summary, these two studies have reflected a tip of the iceberg, among the numerous factors that may affect the immediate and longer-term outcomes after angioplasty ± stenting therapy in symptomatic ICAD, which warrants more clinical studies and *in vitro* investigations.

Volumes I and II of this Research Topic of ICAD studies examine the understudied pathology, pathophysiology, imaging, treatment, and prognosis of ICAD and ICAD-related stroke or TIA. In light of the contributions made by these articles, we continue to advocate for more research in this field, including more accurate assessment and more effective treatment of patients, with the aim of lowering the residual risk of stroke recurrence, and achieving better functional outcomes and quality of life in increasingly affected patients in this aging world.

## Author contributions

XL: Conceptualization, Writing—original draft. SP: Writing—review & editing. JL: Writing—review & editing. AA-C: Writing—review & editing. DL: Supervision, Writing—review & editing.
